# Outcome of surgery for laryngotracheal stenosis in grade III and IV subglottic stenosis

**DOI:** 10.1186/1749-8090-8-S1-O217

**Published:** 2013-09-11

**Authors:** HR Davari

**Affiliations:** 1 General Thoracic Surgery, Tehran University of Medical Sciences, Tehran, Iran

## Background

Treatment for laryngotracheal stenosis is technically challenging and no therapeutic algorithm exists.

## Methods

Thirteen patients with laryngotracheal stenosis were treated. Ten were males and 3 females with an age range of 4–60 years .The cause of airway stenosis was prolong intubation in 10, blunt trauma injury and idiopathic subglottic stenosis in 2 and 1consequence. Preoperative assessment included bronchoscopy, neck and chest CT scan to determine the extension of stenosis. The upper margin of the stricture was 3 mm to 1.0 cm. below the vocal cords; the stenotic segment extended from 3 to 6 cm. All patients except one had tracheostomy for a long period. Two patients had failed previous resections. In 10 patients dilatation and insertion of Montgomery T tube was the initial procedure. Pearson's technique was used for laryngotracheal resection. Suprahyoid laryngeal release was performed in 8. Montgomery T tube were placed in 12 and left in place for 1 year.

## Results

Decanulation was done with success in all except 2 in first attempt. Circumferential granulation in one patient was excised and reinsertion of T tube for another 6 months was associated with successful decanulation. A kid who had resection at age 6 with poor compliance came back early to re-tracheostomy. He re-operated at age 14 with successful final decanulation. Endoscopic Laser and / or APC were used in 2 patients after T tube decanulation.

## Conclusion

Surgical management of laryngotracheal stenosis is the treatment of choice. However, primary surgery is not always feasible. A consequence or combined approach should considered.

